# Sonodynamic Excitation of Rose Bengal for Eradication of Gram-Positive and Gram-Negative Bacteria

**DOI:** 10.1155/2013/684930

**Published:** 2012-12-19

**Authors:** Faina Nakonechny, Michael Nisnevitch, Yeshayahu Nitzan, Marina Nisnevitch

**Affiliations:** ^1^Department of Chemical Engineering, Biotechnology and Materials, Ariel University Center of Samaria, 40700 Ariel, Israel; ^2^The Mina and Everard Goodman Faculty of Life Sciences, Bar-Ilan University, 52900 Ramat-Gan, Israel

## Abstract

Photodynamic antimicrobial chemotherapy based on photosensitizers activated by illumination is limited by poor penetration of visible light through skin and tissues. In order to overcome this problem, Rose Bengal was excited in the dark by 28 kHz ultrasound and was applied for inactivation of bacteria. It is demonstrated, for the first time, that the sonodynamic technique is effective for eradication of Gram-positive *Staphylococcus aureus* and Gram-negative *Escherichia coli*. The net sonodynamic effect was calculated as a 3-4 log_10_ reduction in bacteria concentration, depending on the cell and the Rose Bengal concentration and the treatment time. Sonodynamic treatment may become a novel and effective form of antimicrobial therapy and can be used for low-temperature sterilization of medical instruments and surgical accessories.

## 1. Introduction

The field of photodynamic antimicrobial chemotherapy (PACT) is currently under intense investigation and is showing promising prospects as an alternative to antibiotic treatment in view of increasing widespread bacterial resistance to antibiotics [1, 2]. A classical scheme of PACT includes excitation of low toxic components—photosensitizers (PS) by visible light, when light-activated PS molecules transfer energy to molecular oxygen, which results in the production of reactive oxygen species that in turn cause irreversible damage to cellular components [1, 3–7]. The list of PS includes a wide spectrum of compounds, such as porphyrins, phenothiaziniums, phthalocyanines, hypocrellin derivatives, squaraine derivatives (squaraine dyes, squaric acid derivatives), boron dipyrromethene derivatives, and chlorine derivatives [4, 6, 8]. PACT has been extensively studied as a strategy against both Gram-positive and Gram-negative bacteria [9, 10], including antibiotic-resistant species [11] and appears to occupy a niche for control of oral [12] and other localized infections [11]. It should be noted that no development of bacterial resistance to PS has been reported to date from the numerous studies on the effect of PACT against different microorganisms [13, 14].

The major disadvantage of PACT is the limited tissue penetration of external light. Despite advances in the development of light source devices for phototherapy [15, 16] and the clinical use of PACT in dermatology [5, 17], treatment of internal body tissues remains limited to invasive procedures. Only a few attempts to develop alternative means of PS activation have been reported to date. We [18–20] and others [21] developed an approach in which the external light source was replaced by chemiluminescent light emitted during the course of a chemical reaction. We used the chemiluminescent oxidation of luminol, in which the release of light energy was achieved without electrical or thermal input in the course of *in situ* conversion of molecular oxygen to superoxide ions. This technology, called chemiluminescent photodynamic antimicrobial therapy (CPAT), was shown to be effective against Gram-negative *E. coli* and Gram-positive *S. aureus* (both methicillin-sensitive and methicillin-resistant strains) [19, 20]. Our data showed that CPAT was almost as effective as PACT. CPAT can therefore compete with PACT in eradicating a wide range of Gram-positive and Gram-negative bacteria, including antibiotic-resistant strains. CPAT may thus become a novel antimicrobial therapeutic strategy and might be applicable for internal infections that are difficult to target and treat using traditional PACT. 

Another interesting technique of PS activation that does not use an external light source is sonodynamic excitation by ultrasound. Sonodynamic therapy (SDT) based on ultrasound-induced cytotoxicity of compounds called sonosensitizers has already been studied for cancer cell inhibition. The sonosensitizers include widely used anti-cancer drugs such as bleomycin, adriamycin, amphotericin B, mitomycin C, daunomycin, diaziquone, and 5-fluorouracil [22], as well as several PS, such as hematoporphyrin, photofrin, mesoporphyrin, protoporphyrin, pheophorbide-a, ATX-70 (7,12-bis(1-decyloxyethyl)-Ga(III)-3,8,13,17-tetramethyl-porphyrin-2,18-dipropionyl diaspartic acid), Rose Bengal (RB), zinc(II)-phthalocyanine, and some others [22–29].

Generally speaking, the term SDT can be used for all nonthermally related therapeutic ultrasound applications, ranging from induction of apoptosis when combined with chemotherapy to ultrasound therapy. However, most authors use the term SDT for ultrasonic activation of drugs for cancer therapy [30]. The exact mechanism of SDT has not been entirely elucidated. Furthermore, it is assumed that there is no universal mechanism for synergism between ultrasound and drugs, such that different classes of sonosensitizers can be activated in the dark by different mechanisms [22, 31]. The biological effects of SDT are associated with one of three different mechanisms: heat, mechanical effects, and acoustic cavitation [32]. These effects depend on the intensity and frequency of the ultrasound: high intensity sonication leads to heat production, whereas low frequency treatment causes cavitation. Exposure of biological tissues to ultrasound can result in structural and/or functional changes of cells [32]. Mišík and Riesz believe that biological effects of SDT may be expressed due to one or two combinations of several factors: thermal effects (absorption and dissipation of ultrasound energy), cell membrane permeability changes and/or cell membrane rupture, and free radical effects [31]. The data obtained in their study show that photosensitizers (e.g., porphyrins) can be sonosensitized according to the following scheme: a drug undergoes pyrolysis inside collapsing cavitation bubbles or in the heated gas-liquid interface, forming free radical intermediates which react with dissolved oxygen to form peroxyl radicals, and the latter attack cellular sites due to their ability to diffuse to significant distances [31].

Other authors explain the sonodynamic effect of porphyrins by electronic excitation of the molecules by sonoluminescence, that is, light flashes produced during the course of acoustic cavitation in liquids generated with ultrasound energy without application of external illumination. Sonoluminescence initiates photochemical processes resulting in the formation of cytotoxic singlet oxygen [22, 29, 33]. 

A large number of serious studies are dedicated to SDT of cancer cells, and to the best of our knowledge, this technique has never been applied for eradication of bacteria. In 2009, Ma et al. [34] hypothesized that ultrasound may be exploited for treatment of infectious bacterial and viral diseases and proposed a new concept of sonodynamic antimicrobial chemotherapy (SACT) as a promising novel antimicrobial strategy. However, this hypothesis has not been proven experimentally. 

In the present work it is demonstrated, for the first time, that SACT can indeed be realized. We show that Gram-positive *S. aureus* and Gram-negative *E. coli* can be eradicated by RB activated with ultrasound in the dark.

## 2. Materials and Methods

### 2.1. Bacterial Growth

Cultures of *Staphylococcus aureus* (ATCC 25923) and *Escherichia coli* (ATCC 10798) were grown on brain-heart agar (BHA, Acumedia, USA) for 24 h, then were transferred into brain-heart broth (BH, Acumedia, USA) and were grown at 37°C and at a 170 rpm speed of shaking up to concentration of 10^9^ CFU mL^−1^, centrifuged for 5 min at 10,000 rpm and diluted by sterile 0.05 M PBS, pH 7.5, to concentrations of 106–109 CFU mL^−1^.

### 2.2. Solutions of PS

Stock aqueous solutions of RB and Methylene Blue (MB) (Sigma-Aldrich, USA) were prepared in 0.016 mM and 0.013 mM concentrations, respectively, in PBS and filtered by sterile filtration through 0.22 *μ*m membranes (Pall Corporation, USA). Visible spectra of the PS were registered using UV-Visible Spectrophotometer Cary 50 Bio (Varian, Australia).

### 2.3. Testing of SACT

**Figure 1:**
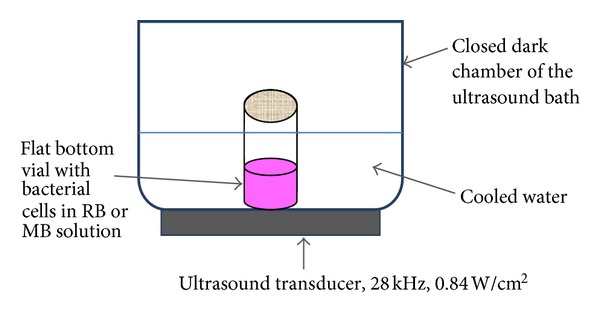
The scheme for the SACT experiments. 2.5 cm diameter flat-bottomed vials with bacterial suspensions in RB or MB solutions were treated with 28 kHz ultrasound at an intensity of 0.84 W cm^−2^ in an ultrasonic bath in the dark.

PS solutions were added to 10 mL portions of diluted *S. aureus* or *E. coli* in flat-bottom 2.5 cm diameter vials (solution height in the vials was 2 cm) and incubated for 15 min in the dark. Vials were held in a plastic holder tight to the bottom of an ultrasonic bath WUG-AO2H (Wise Clean Company, Korea) at 10°C and sonicated for 1-2 h at an ultrasound frequency of 28 kHz and an intensity of 0.84 W cm^−2^ (Figure 1). Strict conditions were maintained in order to prevent any external illumination during the experiments. 100 *μ*L bacteria samples were diluted in several decimal dilutions and were spread over BHA plates with a Drigalsky spreader. The plates were incubated at 37°C overnight and CFU (colony-forming units) were counted taking dilutions into account. In control experiments bacterial cultures were tested in the absence of PS without sonication, in the absence of PS under sonication and in the presence of PS without sonication.

### 2.4. Testing of PACT

PACT experiments were carried out by illumination of bacteria cultured as described above in liquid BH medium, mixed with solutions of MB in glass test-tubes, incubated for 15 min in the dark and illuminated for 30 min at room temperature under temperature control by an 18 W white luminescent lamp placed at a distance of 15 cm from the tubes. Light intensity was 10.6 klux and a fluence rate was 1.6 mW cm^−2^. Light intensity was measured by a LX-102 Light-meter (Lutron, Taiwan). Samples were not sonicated in the PACT experiments.

### 2.5. Statistical Methods

The results obtained from at least 3 independent experiments fulfilled with duplicates were statistically analyzed by Anova single factor or by Anova two-factor analyses. The difference between the results was considered significant if the *P* value was less than 0.05.

## 3. Results and Discussion

Two types of cells were chosen for primary evaluation of the suitability of SDT for eradication of bacteria—*E. coli* for representing Gram-negative bacteria and *S. aureus* as a member of the Gram-positive group. Two compounds, RB and MB, were selected as potential sonosensitizers. These compounds are well known for their photodynamic activity against the chosen bacteria [19, 35–39], and what is more, sonodynamic anticancer activity of RB has been reported in several works [26, 40]. The sonodynamic activity of MB has not been studied, to the best of our knowledge, but several authors believe that photosensitizers are generally good candidates for exhibiting sonodynamic properties [22, 28]. RB and MB have good solubility in water, and this fact enabled building an experimental scheme based on their free forms in aqueous solutions.

The experiments for testing sonodynamic antibacterial properties of RB and MB were held in the dark in an ultrasonic bath with cold water (Figure 1). Pieces of ice were periodically added to the bath in order to compensate for heating of the water during the sonication procedure. All operations were performed in the dark in order to exclude any possibility of photodynamic excitation of the examined compounds. The following control experiments were carried out in all series in the dark: incubation of bacteria without sonication in the absence of RB and MB, incubation of bacteria without sonication in the presence of RB or MB, and sonication of bacteria in the absence of RB and MB. Various concentrations of the bacterial cultures were added to buffered aqueous solutions of RB or MB in flat vials and placed on the bottom of the ultrasound bath for sonication (Figure 1). The control vials, which were not sonicated, were incubated under similar conditions outside the bath in the dark.

**Figure 2:**
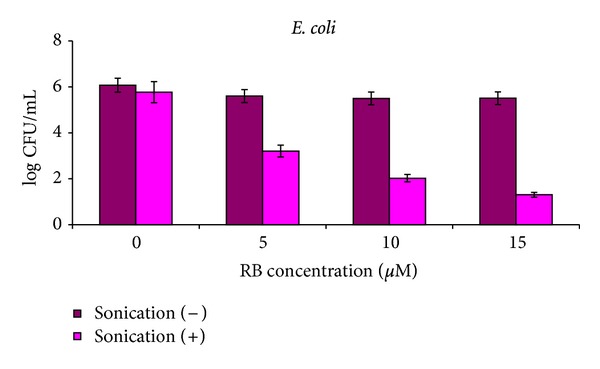
SACT effect of RB on *E. coli.* Cells at initial concentration of 10^6^ CFU mL^−1^ were incubated with 0–15 *μ*M RB in an ultrasonic bath for 1 h in the dark. Controls: not sonicated bacterial cultures with or without PS. After the treatment, bacterial samples were serially diluted in 10-fold dilutions and evenly spread over BHA plates with a Drigalsky spreader. Plates were incubated at 37°C overnight and CFU were counted.

**Figure 3:**
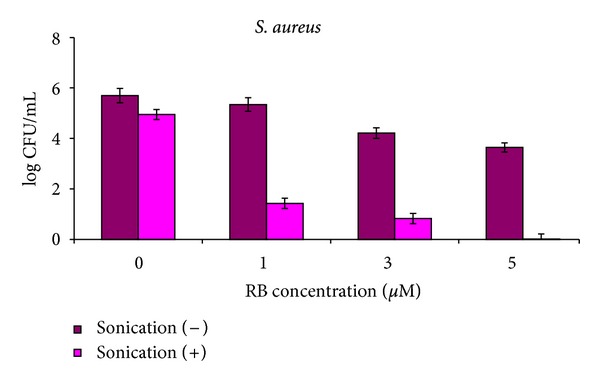
SACT effect of RB on *S. aureus.* Cells at initial concentration of 10^6^ CFU mL^−1^ were incubated with 0–5 *μ*M RB in an ultrasonic bath for 1 h in the dark. Controls: not sonicated bacterial cultures with or without PS. After the treatment, bacterial samples were serially diluted in 10-fold dilutions and evenly spread over BHA plates with a Drigalsky spreader. Plates were incubated at 37°C overnight and CFU were counted.

The results of the SACT experiments demonstrating a sonodynamic effect of RB on *E. coli* are presented in Table 1 and Figure 2, and on *S. aureus* in Table 2 and Figure 3. When treated for 1 h, both *E. coli* and *S. aureus* bacteria were inhibited by RB under each tested concentration, and an increase in RB concentration caused higher suppression. Sonication of the cultures in the absence of RB led to a moderate decrease in cell concentration: sonication of cultures at a high initial bacterial concentration of *E. coli* (10^9^ CFU mL^−1^) resulted in a *ca*. 2 log_10_ decrease in the number of CFU (Table 1), whereas sonication at a low initial concentration (10^6^ and 10^7^ CFU mL^−1^) had no effect on the vitality of the bacteria when compared to the untreated control cells (*P* value = 0.322). Sonication of *S. aureus* not treated by RB resulted in a *ca*. 1 log_10_ decrease in the bacterial concentration for all initial bacterial cell concentrations (Table 2). Incubation of both bacteria in the absence of RB without sonication did not change the concentration of viable cells in all cases (Tables 1 and 2). The dark effect of RB, registered in nonsonicated series, depended on the initial cell concentration: for *E. coli* the maximal dark effect was *ca*. a 1 log_10_ decrease (*P* value = 0.023) in CFU obtained for 15 *μ*M RB at initial concentration of 10^9^ CFU mL^−1^ (Table 1). This effect was even smaller and actually, insignificant, in the case of initial concentrations of 10^6^ and 10^7^ CFU mL^−1^ for all RB concentrations—a 0.1–0.6 log_10_ decrease in the *E. coli* vitality compared to the untreated control cells (*P* values = 0.40–0.72). For *S. aureus* the maximal dark effect was higher than that for *E. coli*—at 5 *μ*M RB for all initial concentrations the vitality of *S. aureus* cells decreased by 1.5–2.5 log_10_ in comparison with untreated cells (*P* values = 0.0238) (Table 2). Actually, in no case did application of either ultrasound or RB separately result in high suppression of the tested bacteria. Simultaneous use of RB and ultrasound in the dark showed quite different results. Inhibition of the treated by 15 *μ*M RB *E. coli* cells was expressed by a 4–4.7 log_10_ decrease in CFU compared to the untreated samples (*P* value = 0.041), (Table 1), and the highest eradication rate was achieved for an initial bacterial concentration of 10^6^ CFU mL^−1^ treated by 15 *μ*M RB, where the remaining *E. coli* concentration dropped to only 20 CFU mL^−1^ (Figure 2). *S. aureus* was even more sensitive to the sonodynamic treatment. The bacteria were suppressed by 3.5–6 log_10_ compared to the untreated samples (*P* values = 0.0078–0.018) at all initial cell concentrations (Table 2), and treatment by 5 *μ*M RB at an initial concentration of 10^6^ CFU mL^−1^ led to the most profound eradication of *S. aureus* (Figure 3). Taking into consideration separate dark and ultrasonic effects, we can conclude that the net contribution of sonodynamic treatment in the dark was *ca*. a 3 log_10_ decrease in CFU. In the cases of both bacteria, application of SACT led to the antibacterial cytotoxic effect.

**Figure 4:**
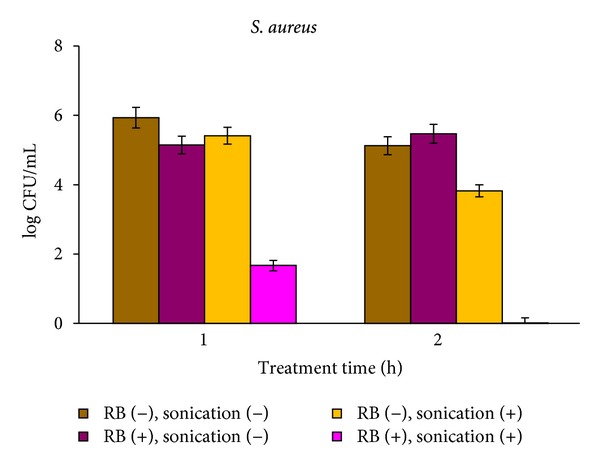
Effect of treatment time on SACT activity of RB*. S. aureus* cells at an initial concentration of 10^7^ CFU mL^−1^ were incubated with 5 *μ*M RB in an ultrasonic bath for 1 and 2 h in the dark. After the treatment, bacteria were tested by viable count.

Prolonging the sonication caused a total bacterial suppression even at higher initial cell concentrations. *S. aureus* at an initial concentration of 10^7^ CFU mL^−1^ and sonicated for 2 h in the presence of 5 *μ*M RB was deeply eradicated, whereas after a 1 h treatment the number of viable cells was 50 CFU mL^−1^ (Figure 4). 

**Figure 5:**
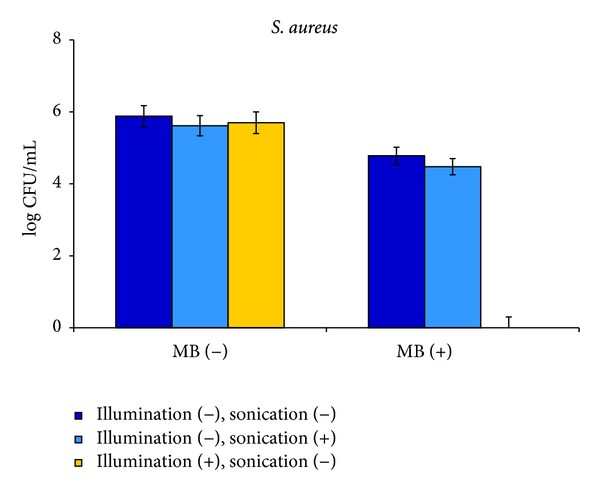
SACT and PACT effect of MB on *S. aureus.* Cells at 10^6^ CFU mL^−1^ concentration were incubated with 30 *μ*M MB in ultrasonic bath for 1 h in the dark. In PACT experiments, the cells were illuminated for 0.5 h with 1.6 mW cm^−2^ white light under the same conditions but without sonication. After the treatment, bacteria were tested by viable count.

Another PS previously shown to be very effective in photodynamic eradication of the bacteria examined in the present study [19, 20, 38, 39], namely MB, was tested under SACT conditions. *S. aureus* was studied at two initial concentrations of 10^9^ and 10^6^ CFU mL^−1^. MB demonstrated a low dark effect in the absence of sonication and the concentration of *S. aureus* cells decreased by 1.2 log_10_ at both initial cell concentrations. At initial bacterial concentration of 10^9^ CFU mL^−1^ sonication in the absence of MB caused a 1.4 log_10_ decrease in CFU, and in the presence of MB—a 1.7 log_10_ reduction in the *S. aureus* concentration. The difference between these data was insignificant (*P* value = 0.39). In the case of initial cell concentration of 10^6^ CFU mL^−1^ the difference was even less (Figure 5) and reached a 0.26 log_10_ decrease in the absence of MB and a 0.3 log_10_ decrease in the presence of MB (*P* value = 0.76) The results of the control experiments were very similar to those obtained in the RB study (Table 2), but in contradistinction to RB, MB was not found to exhibit any sonodynamic activity. Additional control experiments on the photodynamic activity of MB were performed in order to rule out possible occasional inactivity of the batch of MB used for *S. aureus* eradication. *S. aureus* illuminated by white light in the absence of MB was practically unaffected, whereas the bacteria were totally eradicated in the presence of MB (Figure 5).

Inefficiency of MB in the SACT experiment was quite surprising for us and it can be assumed that not all the PS can be excited by ultrasound. Probably, sensitivity of PS to sonication depends on the structure of the molecules, especially on their hydrophoby and electrical charge. Undoubtedly, for more profound investigation of this issue additional studies should be carried out, including variation in PS, in their concentrations and application of ultrasound in a wide range of intensities. 

We have previously examined the effect of free RB on *S. aureus* under photodynamic activation [38, 39] and can now compare PACT with SACT by this compound for the same initial bacterial concentration of 10^7^ CFU mL^−1^. As was shown in [39] total eradication of cells under PACT conditions took place at 1 *μ*M RB excited by white light at a fluence rate of 1.6 mW cm^−2^ applied for half an hour, whereas the same result under the SACT regime in dark conditions was achieved at 5 *μ*M RB activated by ultrasound applied for two hours at an intensity of 0.84 W cm^−2^ and a frequency of 28 kHz. The difference between PACT and SACT was even more drastic for MB. As demonstrated in the present work, *S. aureus* was totally eradicated when activated by light at the same MB and cell concentrations and was almost unaffected when excited by ultrasound (Figure 5). These data demonstrate that overall, PACT is a more effective tool than SACT, but the efficiency of the latter can probably be increased by application of ultrasound at higher intensities or at different frequencies, thus transforming this technique into a practical and convenient tool for bacterial suppression under dark conditions. This issue needs to be investigated further.

**Figure 6:**
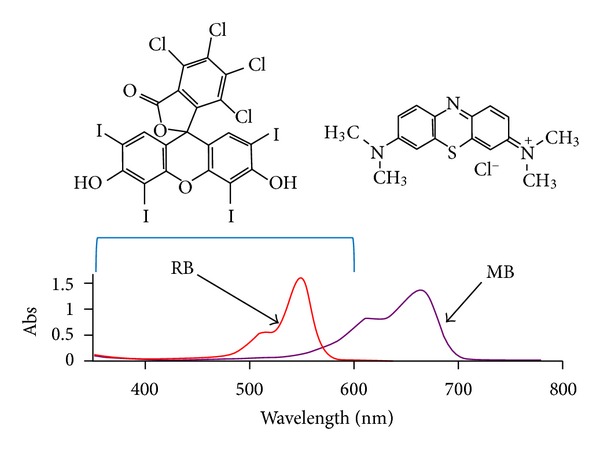
Visible absorption spectra of 0.016 mM RB and 0.013 mM MB aqueous solutions. Structures of RB and MB are shown above the spectra.The region of maximal emission intensity of sonoluminescence in wateris designated by a blue bracket.

The obtained data provide indirect evidence that the mechanism of SACT is probably based on sonoluminescent excitation of PS. Sonoluminescent light has a broadband spectrum from 200 to 700 nm, but the maximum emission intensity of sonoluminescence in water lies between 250 and 600 nm [41]. This emission range correlates well with the absorbance spectrum of RB, but has almost no overlap with the spectrum of MB (Figure 6), thus affording an explanation for the high rate of RB activation by SACT and negligible sonoexcitation of MB. 

Figures 2 and 3 demonstrate that the Gram-positive *S. aureus* responded to the sonodynamic treatment with RB much better than the Gram-negative *E. coli*, where the eradication rates were higher at lower applied RB concentrations for all initial cell concentrations. The same tendency was observed for PACT [38, 39] and was explained by differences in the complex molecular and physico-chemical structure of the cell membranes of the two bacterial groups [42]. The initial cell concentration played a crucial role in the process of bacterial eradication by SACT. For all examined concentrations of RB the SACT effect was more pronounced for low initial bacterial concentrations and less exhibited for concentrated bacterial suspensions. The highest eradication of both bacteria was achieved when cells at low concentration were treated by PS at high concentration (Tables 1 and 2). Probably SACT effect depends on PS dose to bacterial concentration ratio. Analogous phenomenon of different effect of PS on bacterial cells at various concentrations was registered earlier in PACT experiments [39] and it was shown that at low concentrations bacteria showed stronger susceptibility for PACT treatment. 

As in the case of cancer treatment, combating bacterial infections *in vivo* can be more effective and with fewer side effects when targeted to the site of infection. In addition to known methods of targeting drugs to cells, including antibody, protein or peptide, liposomal, and magnetic targeting [43–50], use of ultrasound affords a direct and exact focusing on the treated site. Nonactivated PS does not harm healthy tissues. Thus, excitation of the PS only after it has reached the treatment site will prevent systemic toxic effects during delivery of the drug to the treatment site. Such targeted treatment was carried out on cancer cells in mice by Yumita et al. [29] and Umemura et al. [51] and resulted in cessation of tumor growth [51] or even in destruction of tumor tissue [29]. The same targeting scheme can undoubtedly be applied for antibacterial treatment. 

In addition to future potential therapeutic applications of SACT, a method for cold sterilization of medical instruments and surgical accessories could be proposed. A simple addition of sonosensitizers, for example of RB, to the aqueous phase in the ultrasonic bath will significantly improve and increase the effectiveness of the sterilization process. 

## 4. Conclusions

The present work shows for the first time, that sonodynamic activation of RB causes eradication of *E. coli* and *S. aureus* in the dark. The described novel SACT technique has good prospects for becoming an effective targeted tool for combating internal infections and for cold sterilization of medical instruments.

## Figures and Tables

**Table 1:** SACT effect of RB on *E. coli *cells at various initial concentrations.

log_10_ *C*_0_	RB concentration, *μ*M							
	0		5		10		15	
	+*	−	+	−	+	−	+	−
6	5.8	6.1	3.2	5.6	2.0	5.5	1.3	5.5
7	7.0	6.9	5.6	6.8	4.5	6.3	3.7	6.3
9	7.1	9.0	6.8	9.0	6.0	8.2	5.0	8.1

*Plus (+) designates SACT treated samples and minus (−) designates controls. Treatment time was 1 h. *C*_0_: initial cell concentration, CFU mL^−1^.

**Table 2:** SACT effect of RB on *S. aureus *cells at various initial concentrations.

log_10_ *C*_0_	RB concentration, *μ*M							
	0		1.5		3		5	
	+*	−	+	−	+	−	+	−
6	5.0	5.7	1.4	5.3	0.8	4.2	0.01	3.6
7	6.0	6.9	3.7	5.8	2.9	5.6	1.7	5.6
9	7.9	8.9	6.6	8.5	6.3	7.8	5.4	7.5

*Plus (+) designates SACT treated samples and minus (−) designates controls. Treatment time was 1 h. *C*_*o*_: initial cell concentration, CFU mL^−1^.
